# Viral-Mediated Knockdown of Nucleus Accumbens Shell PAC1 Receptor Promotes Excessive Alcohol Drinking in Alcohol-Preferring Rats

**DOI:** 10.3389/fnbeh.2021.787362

**Published:** 2021-12-03

**Authors:** Margaret A. Minnig, Tayun Park, Maria Echeveste Sanchez, Pietro Cottone, Valentina Sabino

**Affiliations:** Laboratory of Addictive Disorders, Department of Pharmacology and Experimental Therapeutics, Boston University School of Medicine, Boston, MA, United States

**Keywords:** ethanol, self-administration, alcohol use disorder (AUD), PAC1R, pituitary adenylate cyclase activating polypeptide (PACAP), compulsive, addiction, neuropeptide

## Abstract

Alcohol use disorder (AUD) is a chronic, relapsing disorder whose genetic and environmental susceptibility components are not fully understood. Neuropeptidergic signaling has been repeatedly implicated in modulating excessive alcohol drinking, especially within sub-regions of the striatum. Here, we investigated the potential involvement of the selective receptor for pituitary adenylate cyclase-activating polypeptide (PACAP), PAC1R, in the nucleus accumbens shell (NAcc Shell) in excessive alcohol drinking in alcohol-preferring rats, an established animal model of the genetic propensity for alcoholism. Scr:sP alcohol-preferring rats were trained to operantly self-administer alcohol and then either an AAV virus short-hairpin RNA (shRNA) targeted to knockdown PAC1R, or an AAV control virus were microinfused into the NAcc Shell. NAcc Shell PAC1R shRNA knockdown virus was confirmed to significantly decrease PAC1R levels in the NAcc Shell. The effects of NAcc Shell PAC1R shRNA knockdown on ethanol self-administration were investigated using a Fixed Ratio (FR) 1 and a Progressive Ratio (PR) schedule of reinforcement. The effect of PAC1R knockdown on self-administration of an alternative reinforcer, saccharin, was also assessed. The results showed that the reduction in PAC1R in the NAcc Shell led to excessive ethanol drinking, increased preference for ethanol, and higher motivation to drink. NAcc Shell PAC1R shRNA knockdown did not comparably increase saccharin self-administration, suggesting selectivity of action. These data suggest that NAcc Shell PAC1R may serves as a “brake” on alcohol drinking, and thereby the loss of function of PAC1R leads to excessive alcohol consumption. Therefore, the PACAP/PAC1R system may represent a novel target for the treatment of AUD.

## Introduction

Alcohol use disorder (AUD) is a chronic, relapsing disorder that affects over 14 million adults in the US (Substance Abuse and Mental Health Services Administration (SAMHSA), 2019), and is estimated to be responsible for 5.3% of global deaths (Shield et al., 2020). Both environmental and genetic factors contribute to AUD susceptibility, with the hereditability estimated at 50–60% of the total phenotypic variability (Reilly et al., 2017). Animal models of hereditary preference for alcohol are an invaluable tool for investigations into the genetic component of AUD (Ciccocioppo and Hyytia, 2006; Crabbe, 2014; Gessa, 2016). One such model, a line of alcohol-preferring rats genetically selected for heavy alcohol consumption, the Sardinian alcohol-preferring rats, was utilized in this study (Colombo et al., 2006; Sabino et al., 2007, 2011, 2013a,b; Blasio et al., 2015).

The neuropeptide pituitary adenylate cyclase activating polypeptide (PACAP) belongs to the neuropeptide superfamily that includes vasoactive intestinal peptide (VIP), growth hormone-releasing hormone (GHRH), and secretin (Sherwood et al., 2000). PACAP has been implicated in a large variety of homeostatic systems within the body, including energy metabolism, food intake, body temperature, neuronal survival, reproduction (Gray et al., 2002; Inglott et al., 2011; Resch et al., 2011, 2014; Iemolo et al., 2015; Ross et al., 2018), as well as in the body’s response to stress and in several neuropsychiatric disorders (Hammack et al., 2009, 2010; Ressler et al., 2011; Dore et al., 2013; Mustafa et al., 2015; Seiglie et al., 2019; Ross et al., 2020; Varodayan et al., 2020, reviewed in Hammack and May, 2015; Lutfy and Shankar, 2019).

The PACAP system has recently been implicated in substance use disorders more generally, and in AUD specifically (for review, see Gargiulo et al., 2020; Stojakovic et al., 2020). Acute ethanol leads to an increase in the mRNA levels of the PACAP-selective receptor, PAC1R, via the Receptor for Activated C Kinase 1 (RACK1) scaffolding protein in cell lines (He et al., 2002). PACAP modulates the hypothermic effects of alcohol (Szabó et al., 1998; Tanaka et al., 2004), and tolerance to acute ethanol-induced ataxia (Moore et al., 1998). Recent reports provide evidence that PACAP-27 and PACAP-38 administration modulate home cage alcohol drinking (Gargiulo et al., 2021), and that ethanol exposure increases PACAP expression in the paraventricular nucleus of the thalamus (Gupta et al., 2018) and in the bed nucleus of the stria terminalis (Ferragud et al., 2021). In human studies, a single nucleotide polymorphism of the gene coding for PACAP, ADCYAP1, was linked to higher levels of alcohol intake in a Finnish population of social drinkers (Kovanen et al., 2010), and a variant of PAC1R was found to be associated with problematic alcohol use in women (Dragan et al., 2017).

The nucleus accumbens (NAcc) Shell is a key substrate for the actions of drugs and alcohol, playing a critical role in the establishment of their acute reinforcing effects and in incentive salience (Koob and Volkow, 2010). The NAcc Shell receives input from a number of other limbic and midbrain structures, including the infralimbic (IL) subregion of the medial prefrontal cortex, a projection proposed to be part of a “stop” circuitry in the context of drug and alcohol seeking (Koob and Volkow, 2016). Interestingly, immunoreactivity for PACAP is found in the NAcc Shell, as well as expression of PAC1R (Cauvin et al., 1991; Ghatei et al., 1993; Palkovits et al., 1995; Zhang et al., 2021).

This study aimed to investigate a potential contribution of the PAC1R specifically in the NAcc Shell in alcohol drinking in rats, using a viral vector-mediated knockdown.

## Materials and Methods

### Animals

Subjects in this study were male rats derived from TSRI Sardinian alcohol-preferring rats (Scr:sP, 29–30th generation^1^) which were derived through intra-line breeding at The Scripps Research Institute from sP rats (32nd generations of selective breeding, courtesy Prof. G.L. Gessa, University of Cagliari, Italy). These animals were maintained without further selective breeding at Boston University, and housed in an AAALAC-approved vivarium with a 12 h light-dark cycle. Experiments were performed during the dark cycle. Regular rodent chow and water were available *ad libitum.* All procedures were approved by Boston University Medical Campus Institutional Animal Care and Use Committee and followed National Institutes of Health *Guide for the Care and Use of Laboratory Animals* and *Principles of Laboratory Animal Care* guidelines.

### Drugs

Ethanol (10% *w/v*) and saccharin (0.02% *w/v*) solutions were prepared in tap water using 95% ethyl alcohol and saccharin sodium salt hydrate (Sigma Aldrich, St. Louis, MO), respectively. Adeno-associated viruses (AAV) containing either a custom-designed PAC1R short-hairpin RNA for PAC1R with a green fluorescent protein or only a green fluorescent protein were: AAV1-CAG-rADCYAP1R1-shRNAmir-GFP (“AAV-PAC1R KD,” Vigene Biosciences, Rockville, MD) and AAV1-CAG-GFP (“AAV-CTRL,” Addgene, Watertown, MA).

### Intracranial Surgery

Animals were intracranially injected bilaterally with either an AAV-PAC1R KD or an AAV-CTRL, via a 2 μl, 22-gauge syringe (Hamilton, Reno, NV). Coordinates used for the NAcc Shell injections were (in mm): AP: +1.45, ML: ±2.5 (6-degree angle), DV: −7.1, and a volume of 500 nl per side was infused. For all surgeries, the incisor bar was set to −3.3 mm from the interaural line (flat skull), as per the Paxinos atlas (Paxinos and Watson, 2007).

### Apparatus for Operant Oral Self-Administration

The test chambers used for operant oral self-administration (Med Associates, Inc., St. Albans, VT) were located in sound-attenuating, ventilated cubicles (66 × 56 × 36 cm) (Blasio et al., 2015; Ferragud et al., 2021). Syringe pumps (Med Associates, St. Albans, VT) dispensed ethanol (or saccharin) and water into two stainless steel drinking cups mounted 2 cm above the grid floor in the middle of one side panel. Two retractable levers were located 3.2 cm to either side of the drinking cups. Fluid delivery and operant responses were controlled by microcomputers with 10 ms resolution.

### Fixed Ratio-1 Schedule Self-Administration Procedure

*FR-1 Training:* Rats (*n* = 8/group) were first allowed continuous (24-h/day) home cage two-bottle choice access to ethanol (10% *w/v*, prepared using ethyl-alcohol and tap water) for 1 week, and then moved to limited access (2 h/day) for 4 days. During two-bottle choice training, rats always had access to both ethanol and water. Rats then were allowed two-choice operant self-administration access to ethanol and water for 1–3 overnight sessions (16 h, with food available *ad libitum*) until they reached approximately 100 lever presses for ethanol within a session. Subsequently, animals performed daily self-administration sessions (30 min) until stable responding was reached (<20% variation across three consecutive sessions). Across all sessions, lever presses had no scheduled consequences for 2.01 s after the activation of the pumps to avoid double-lever hits (Sabino et al., 2006, 2009).

*FR-1 following AAV Infusion:* Following stable performing in FR-1 operant sessions as described above, rats were matched based on baseline alcohol intake and body weight into two groups. Either AAV-PAC1R or AAV-GFP virus was infused into NAcc Shell bilaterally as described above. After 3 weeks of viral incubation, a time-course commonly used in rodent brain tissue to ensure sufficient viral infection (Reimsnider et al., 2007; Aschauer et al., 2013), 30 min self-administration sessions resumed for a total of 14 post-injection sessions (2 weeks). The preference for ethanol reported from these self-administration sessions is calculated as (total infusions (ml) for ethanol/[total infusions (ml) for ethanol + total infusions (ml) for water]) × 100.

### Progressive Ratio Schedule Self-Administration Procedure

Following FR-1 self-administration, rats were allowed to self-administer 10% *w/v* ethanol under a PR schedule of reinforcement, where the number of responses required to deliver an ethanol reinforcer increases with successive deliveries [progression: response ratio = 4 × (e^#of  reinforcer ×0.1^) − 3.8, rounded to the nearest integer (Sabino et al., 2011)]. The session began with the completion of the first ratio, with the latency to complete the first ratio set to a maximum of 2 h or when rats had not completed a ratio for 14 min, as previously reported (Gilpin et al., 2008; Sabino et al., 2011). Three responses were required to start the session to avoid unintentional starts (PR schedule: 3, 1, 2, 2, 3, 3, 4, 5, 6, 7, 8, 9, 11, 12, 14, 16, 18, 20, 23, etc.). The last completed ratio was defined as the breakpoint. Responses on the inactive lever were also recorded.

### Fixed Ratio-1 Saccharin Self-Administration Procedure

At the end of the PR ethanol self-administration experiment, rats were allowed to perform self-administration sessions as described above, but with a choice between a saccharin solution (0.02% *w/v*) and water. This concentration was chosen because it maintains responding rates that are the same as 10% *w/v* ethanol. One rat had to be euthanized prior to saccharin intake, reducing the PAC1R KD group to *n* = 7.

### Immunohistochemistry

One hour after the final operant session, rats were deeply anesthetized with isoflurane and perfused transcardially with cold phosphate-buffered saline (PBS), followed by ice cold 4% paraformaldehyde (PFA). The brains were dissected, stored in 4% PFA overnight, and then placed in a 30% sucrose solution for 48 h. Coronal brain sections were cut at 40 μm using a cryostat. Every 6th section through the NAcc Shell (range: +2.28 to +0.84) was collected and used for immunohistochemistry to verify protein knockdown in a semi quantitative manner (non-calibrated to known protein concentration), as done previously (Musatov et al., 2006; Scheimann et al., 2019; You et al., 2019). Following Tris buffered saline (TBS) washes, sodium citrate buffer antigen retrieval was performed at 95 °C for 10 min. Sections were then incubated in a blocking solution for 1 h (5% normal goat serum, 0.2% Triton X-100 in TBS) and then incubated in primary antibodies in blocking solution for 48 hr at 4 °C (rabbit anti-PAC1R, AVR-003, 1:250, Alomone Labs, Jerusalem, Israel; chicken anti-GFP, ab13970, 1:1,000, Abcam, Cambridge, MA; for immunizing peptide validation see Supplementary Figure 1 in Varodayan et al. (2020). After TBS washes, secondary antibody incubation (anti-rabbit AF555, A21429 1:500, Invitrogen, Carlsbad, CA; anti-chicken AF488, 103-545-155 1:500, Jackson ImmunoResearch, West Grove, PA) was performed for 2 h at room temperature. Sections were then mounted and coverslipped with DAPI Hardset mounting medium (Vector Laboratories, Burlingame, CA).

### Quantification of Immunohistochemistry

**FIGURE 1:**
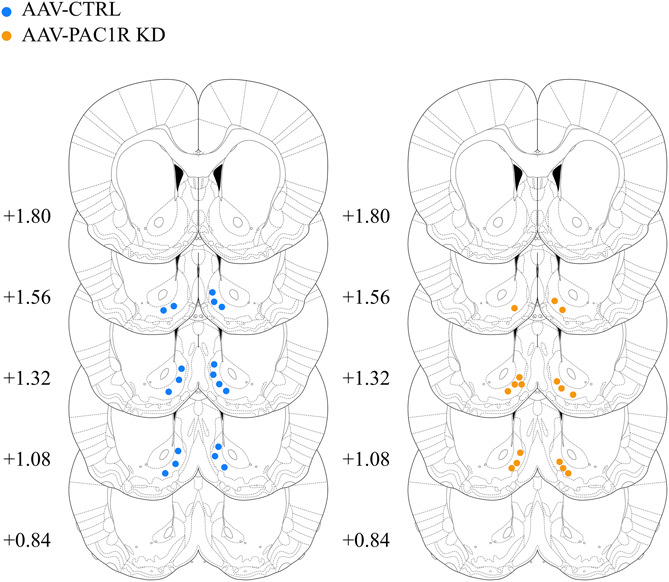
Center of AAV injection sites in AAV-CTRL and AAV-PAC1R KD groups. All injections were restricted to the NAcc Shell.

Representative images of staining were taken at a 20× magnification on an Olympus BX-51 microscope. On a subset of rats (*n* = 6/group), unbiased stereological counts of cells expressing PAC1R, GFP, or both in the NAcc Shell were performed as previously described (Dore et al., 2013; Ferragud et al., 2021). The area was outlined using an Olympus PlanApo N x2 objective with numerical aperture 0.08, and counting was performed with an Olympus UPlanFL N x20 objective with numerical aperture 0.75. Cells were counted by an experimenter blind to experimental group using the Optical Fractionator Workflow module in Stereo Investigator software (MBF Biosciences, Williston, VT). In this workflow, the following parameters were used: the grid frame and counting frame were 250 × 250 μm, the guard zone was 2 μm and the dissector height was 20 μm. The Stereo Investigator software was used to estimate the average mounted thickness of sections, and this value was used to estimate the total volume of the counted region, as well as the total number of PAC1R+ cells. GFP staining of viral injection site was imaged at 2× on an Olympus BX-51 microscope for viral placement verification. One animal was excluded for incorrect placement (unilateral injection). The center of injection sites is included below in Figure 1, all included animals had expression contained to the NAcc Shell.

### Statistical Analysis

Data from FR-1 and PR experiments were analyzed using two-way split plot analyses of variance (ANOVAs), with Group as a between-subjects factor, and Session as a within-subject factor. Pairwise *post hoc* comparisons were made using the Student–Newman–Keuls tests, Student’s *t*-test was used when comparing two groups. Significance was set at *p* ≤ 0.05. The software/graphic packages used were Statistica 7.0, and GraphPad 9.2.

## Results

### NAcc Shell PAC1R shRNA Knockdown Induces Excessive Drinking in Scr:sP Alcohol-Preferring Rats

**FIGURE 2:**
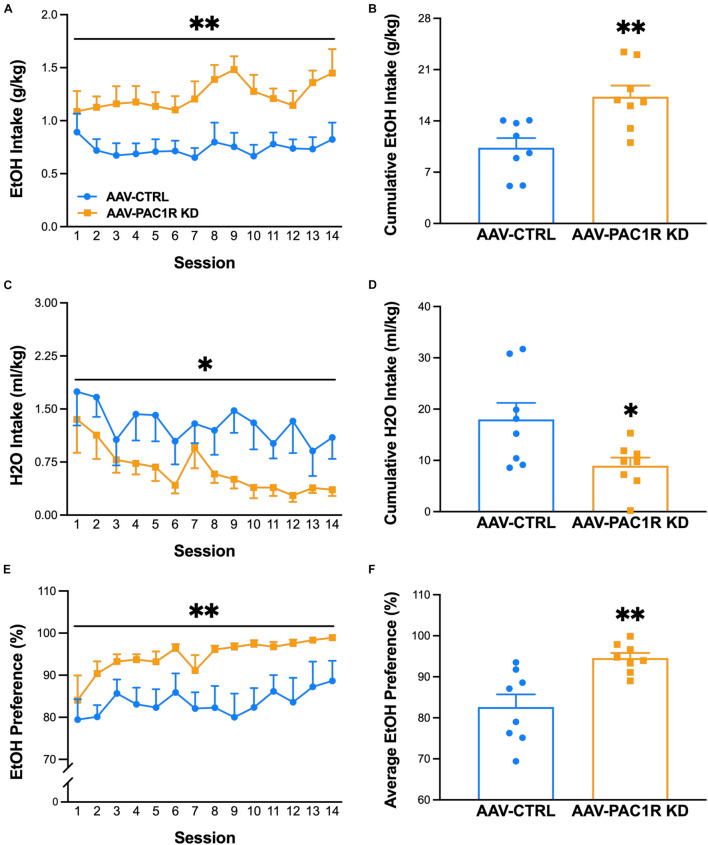
Effect of AAV-mediated short-hairpin virus knockdown of PAC1R in the NAcc Shell in Scr:sP rats on ethanol self-administration in an FR-1 schedule of reinforcement (*n* = 8/group). **(A,B)** Rats with NAcc Shell PAC1R shRNA knockdown (“AAV-PAC1R KD”) drank significantly more ethanol than control AAV rats (“AAV-CTRL”). **(C,D)** NAcc Shell AAV-PAC1R KD rats drank less water in later sessions. **(E,F)** NAcc Shell AAV-PAC1R KD rats also showed a higher preference for ethanol compared to AAV-CTRL rats. Data represent Mean ± SEM. ^∗^*p* ≤ 0.05, ^∗∗^*p* ≤ 0.01.

Following the FR-1 ethanol training sessions, rats were split into two groups, matched for body weight and average ethanol intake over the final three sessions before surgery [baseline: AAV-CTRL group 1.1 ± 0.1 g/kg, AAV-PAC1R KD group 1.0 ± 0.1 g/kg, *t*(14) = 0.07, *p* = 0.95]. Body weight remained relatively stable across sessions, and percent bodyweight change did not differ between groups [AAV-CTRL: 7.7 ± 1.56%, AAV-PAC1 KD: 5.1 ± 1.57%, *t*(14) = 0.26, n.s.]. Scr:sP rats with virally mediated NAcc Shell PAC1R shRNA knockdown drank significantly more ethanol [Figure 2A, Group: *F*(1, 14) = 11.82, *p* ≤ 0.01, Group^∗^Session: *F*(13, 182) = 1.03, n.s.] and displayed a higher ethanol preference [Figure 2E, Group: *F*(1, 14) = 12.69, *p* ≤ 0.01, Group^∗^Session: *F*(13, 182) = 0.27, n.s.], compared to rats infused with a control virus. This higher drinking was evident when comparing the cumulative alcohol intake across all sessions [Figure 2B, *t*(14) = 3.44, *p* ≤ 0.01], and the average preference across all sessions [Figure 2F, *t*(14) = 3.56, *p* ≤ 0.01]. Correspondingly, the AAV-PAC1R KD rats pressed for ethanol a greater number of times per session compared to AAV-CTRL [AAV-CTRL: 37.9 ± 3.51, AAV-PAC1 KD: 56.1 ± 5.46, *t*(14) = 3, 38, *p* ≤ 0.01, data not shown]. NAcc Shell AAV-PAC1R KD rats drank significantly less water than AAV-CTRL rats across sessions [Figure 2C, Group: *F*(1, 14) = 6.25, *p* ≤ 0.05, Group^∗^Session: *F*(13, 182) = 0.48, n.s.], and cumulatively [Figure 2D, *t*(14) = 2.500, *p* ≤ 0.05]. This decreased water drinking did not compensate for the high levels of ethanol intake by NAcc Shell PAC1R shRNA knockdown rats in terms of total fluid intake, which was, therefore, also increased compared to controls [Group: *F*(1, 14) = 9.54, *p* ≤ 0.001, Group^∗^Session: *F*(13, 182) = 0.85, n.s., data not shown].

### NAcc Shell PAC1R shRNA Knockdown Increases Motivation of Scr:sP Rats to Drink Alcohol

**FIGURE 3:**
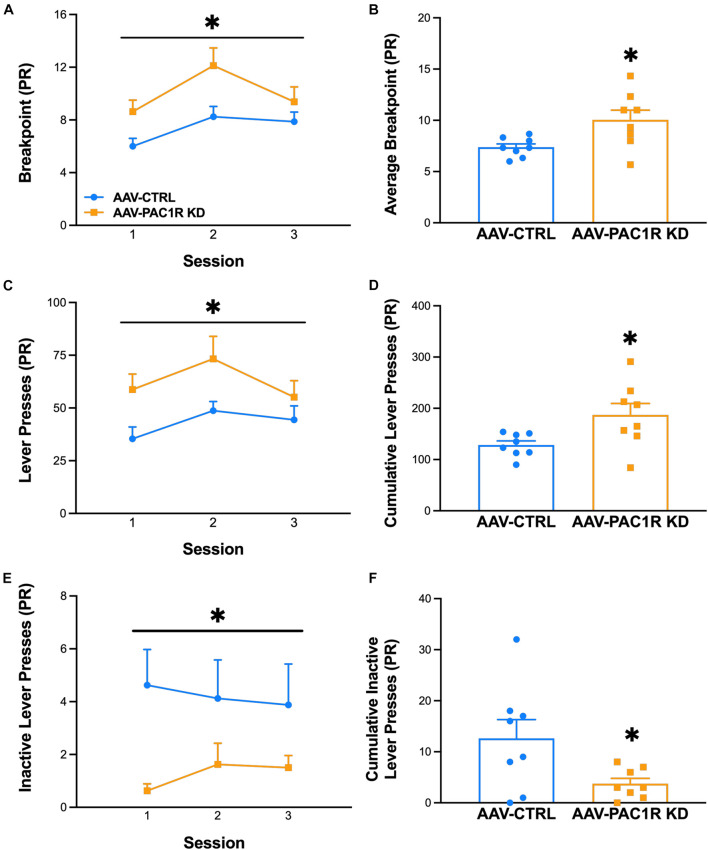
Effect of NAcc Shell PAC1R shRNA knockdown on motivation to drink alcohol in a progressive ratio schedule of reinforcement (*n* = 8/group). **(A,B)** Rats with NAcc Shell PAC1R shRNA knockdown (“AAV-PAC1R KD”) showed a higher breakpoint in the PR schedule, compared to control rats (“AAV-CTRL”). **(C,D)** NAcc Shell AAV-PAC1R KD rats also displayed higher lever presses for ethanol, compared to AAV-CTRL rats. **(E,F)** AAV-CTRL rats pressed the inactive lever more often than NAcc Shell AAV-PAC1R KD rats. Data represent Mean ± SEM. ^∗^*p* ≤ 0.05.

As shown in Figure 3, NAcc Shell PAC1R knockdown increased the motivation to drink ethanol, as measured by breakpoint in a PR schedule of reinforcement [Figure 3A, Group: *F*(1, 14) = 6.95, *p* ≤ 0.05, Group^∗^Session: *F*(2, 28) = 1.24, n.s., Figure 3B, *t*(14) = 2.36, *p* ≤ 0.05]. The total ethanol lever presses were also significantly higher in NAcc Shell PAC1R KD rats, compared to AAV-CTRL rats [Figure 3C: Group: *F*(1, 14) = 6.15, *p* ≤ 0.05, Group^∗^Session: *F*(2, 28) = 0.85, n.s., Figure 3D, *t*(14) = 2.48, *p* ≤ 0.05]. The AAV-CTRL rats pressed the inactive lever more frequently than the AAV-PAC1 KD rats [Figure 3E, Group: *F*(1, 14) = 5.36, *p* ≤ 0.05, Group^∗^Session: *F*(2, 28) = 0.70, n.s., Figure 3F: *t*(14) = 2.32, *p* ≤ 0.05].

### NAcc Shell PAC1R shRNA Knockdown Does Not Affect Saccharin Self-Administration in Scr:sP Rats

**FIGURE 4:**
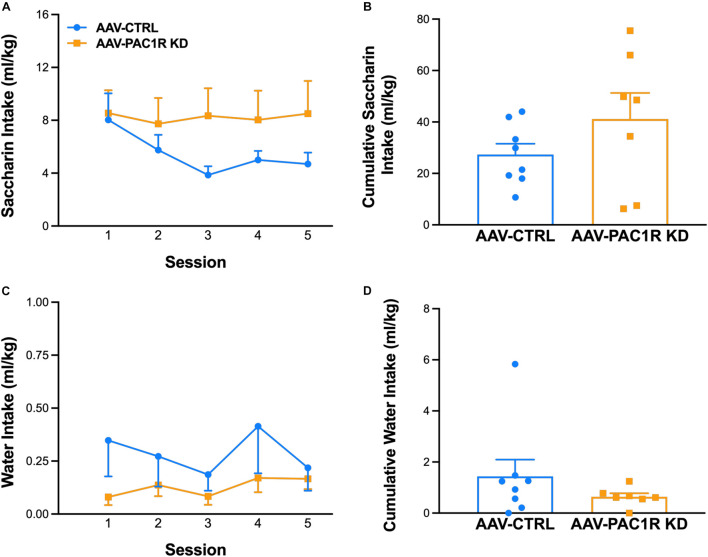
Effect of NAcc Shell PAC1R shRNA knockdown on saccharin self-administration (FR-1 schedule) (*n* = 7–8/group). **(A,B)** Rats with NAcc Shell PAC1R shRNA knockdown (“AAV-PAC1R KD”) and control rats (“AAV-CTRL”) showed no significant differences in self-administration of the alternative reinforcer saccharin, or of the concurrently available water **(C,D)**. Data represent Mean ± SEM.

Saccharin intake was not affected by NAcc Shell PAC1R shRNA knockdown, as shown in Figure 4A [Group: *F*(1, 13) = 1.75, n.s., Group^∗^Session: *F*(4, 52) = 1.83, n.s.]. Cumulative saccharin intake across all session was also unaffected [Figure 4B, *t*(13) = 1.32, n.s.], as well as lever presses for saccharin [AAV-CTRL: 26.8 ± 4.30, AAV-PAC1 KD: 43.3 ± 11.00, *t*(13) = 1.47, n.s., data not shown]. Water intake during saccharin self-administration sessions also did not differ between groups [Figure 4C, Group: *F*(1, 13) = 1.26, n.s., Group^∗^Session: *F*(4, 52) = 0.79, n.s., Figure 4D, *t*(13) = 1.12, n.s.].

### Confirmation of NAcc Shell PAC1R shRNA Knockdown

**FIGURE 5:**
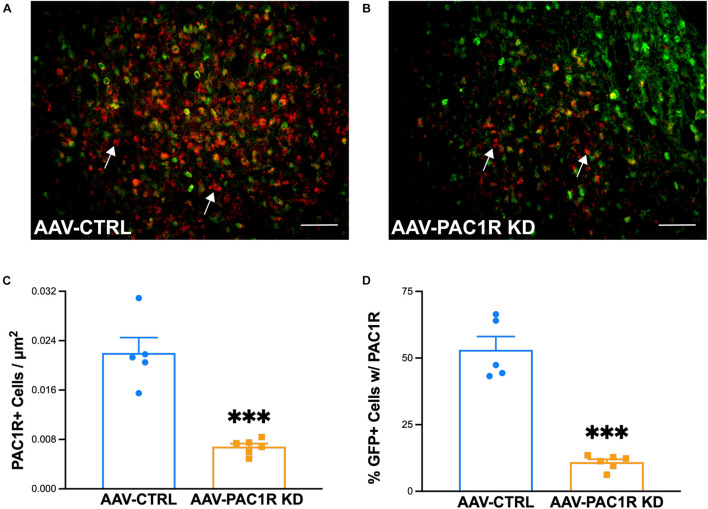
Effect of NAcc Shell PAC1R shRNA knockdown on PAC1R levels in the NAcc Shell of rats (*n* = 6/group). **(A,B)** Representative images of PAC1R (red) and GFP (green) expression in the NAcc Shell of AAV-CTRL and AAV-PAC1R KD rats **(C)** NAcc Shell PAC1R shRNA knockdown rats (“AAV-PAC1R KD”) showed a significant reduction in the number of PAC1R positive cells in the NAcc Shell, compared to AAV controls (“AAV-CTRL”). **(D)** NAcc Shell PAC1R shRNA knockdown also significantly reduced the number of PAC1R positive cells specifically in virally infected cells (GFP-labeled cells). White arrows point to example PAC1R-labeled cells. Scale bars represent 100 μm. Data represent Mean ± SEM. ^∗∗∗^*p* ≤ 0.001.

As shown in Figure 5, NAcc Shell PAC1R knockdown was successful in reducing the number of PAC1R+ cells in the NAcc Shell [Figure 5C, *t*(9) = 6.54, *p* ≤ 0.001]. The number of cells expressing PAC1R that were virally infected (GFP+) was also significantly reduced in AAV-PAC1R KD rats compared to AAV-CTRL [Figure 5D, *t*(9) = 9.00, *p* ≤ 0.001].

## Discussion

The main findings of this study were that NAcc Shell PAC1R knockdown via an AAV-shRNA led to: (1) higher self-administration of, and preference for, alcohol; (2) increased motivation to drink alcohol; (3) no significant change in self-administration of the alternative reinforcer saccharin; and (4) a decrease in PAC1R+ cells as well as in virally infected cells expressing PAC1R in the NAcc Shell, as compared to a control virus infusion. These findings suggest an important role for the PACAP/PAC1R system in the NAcc Shell in the control of alcohol drinking.

The Scr:sP rat line used in this study descends from the original Sardinian alcohol-preferring (sP) rat line, which was selectively bred from Wistar rats for its preference for alcohol, and it represents an established model of hereditary excessive alcohol drinking (Colombo et al., 2006; Sabino et al., 2011, 2013a,b; Blasio et al., 2015).

NAcc Shell shRNA PAC1R knockdown in Scr:sP rats led to significantly higher alcohol drinking and preference for alcohol in an FR-1 schedule of reinforcement, compared to rats with a control virus infusion. NAcc Shell PAC1R knockdown rats drank more and had a higher preference for alcohol compared to control rats beginning on the second and third daily session, respectively, and the two groups maintained this difference in ethanol intake over the course of the 14-day observation period. It should be noted that the control group started with one initial self-administration session similar to the shRNA PAC1R knockdown group following the 3-week AAV incubation period, and then stabilized at a lower level for the rest of the sessions, which is consistent with an alcohol deprivation effect, commonly seen in rats, including alcohol-preferring rats, after a period of abstinence (Heyser et al., 1997; Rodd et al., 2003; Vengeliene et al., 2003; Schroeder et al., 2005; Bell et al., 2006).

Interestingly, NAcc Shell PAC1R knockdown decreased water intake in the later sessions, which can be interpreted as a compensatory behavior for the increased ethanol intake to try and maintain the total fluid intake. The interpretation that water reduction was not a specific effect is supported by the data obtained in the saccharin experiment, where water intake was unaffected by the NAcc Shell PAC1R knockdown. Similar compensatory adjustments in water intake have been previously shown for several other drugs which modulate concurrent alcohol intake (Ericson et al., 1998; Rezvani et al., 2007; Steensland et al., 2007; Suchankova et al., 2013). In addition, we observed that NAcc Shell PAC1R knockdown increased the motivation to drink alcohol in a PR schedule. Under PR reinforcement schedules, ratio requirements increase with subsequent reinforcer deliveries and the influence of local response rates on performance are reduced. NAcc Shell PAC1R knockdown reduced the break point, an objective measure of the effort an animal will expend to obtain a reinforcer that is sensitive both to the subjects’ incentive state and to the reinforcer’s stimulus properties (Walker et al., 2008).

In contrast to alcohol drinking, NAcc Shell shRNA PAC1R knockdown did not alter self-administration of an equally reinforcing saccharin solution. Saccharin is often used as an alternative reinforcing solution to assess the selectivity of the effects of a manipulation on alcohol intake vs. a more general one on all reinforcing substances (Sabino et al., 2009, 2011, 2013b; Valenza et al., 2016; Torruella-Suárez et al., 2020; Benvenuti et al., 2021; Kwok et al., 2021). An alternative interpretation of our findings could be that NAcc Shell shRNA PAC1R knockdown increases the palatability of alcohol, as prior reports have shown that PACAP administration in the NAcc reduces appetitive orofacial responses to sucrose and hedonic eating, although this finding was unique to the NAcc Core (Hurley et al., 2016, 2019). However, this is unlikely due to the similar saccharin self-administration in AAV-PAC1R KD and AAV-CTRL rats. As saccharin is not caloric, it is possible that the NAcc Shell shRNA PAC1R knockdown drives animals to increase caloric intake, although this seems unlikely in light of the fact that PACAP effects on hunger are mediated by hypothalamic regions (Resch et al., 2011, 2014; Hurley et al., 2019). Further, it has been previously reported that PACAP administration in the NAcc Shell does not affect sucrose intake (Gargiulo et al., 2021), further supporting the notion that the NAcc PACAP/PAC1R system plays a selective role in the effects of alcohol. Future studies may directly address the question of whether NAcc Shell PAC1R knockdown increases caloric intake.

PACAP does not appear to be synthetized within the NAcc (mRNA not found locally), and therefore an upstream source of PACAP onto NAcc PAC1R containing neurons is hypothesized (Köves et al., 1991; Masuo et al., 1993; Zhang et al., 2021). Major inputs to the NAcc originate from the medial prefrontal cortex (mPFC), which in the rat is comprised of dorsal (prelimbic; PrL) and ventral (infralimbic; IL) areas (Ongür and Price, 2000; Heidbreder and Groenewegen, 2003), that project to the NAcc Core and Shell, respectively (Barker et al., 2015; Koob and Volkow, 2016; Ma et al., 2020; Zinsmaier et al., 2021). These differential glutamatergic inputs are thought to provide functional specialization to striatal sub-regions (Cox and Witten, 2019), and a “Stop and Go” system has been proposed, wherein the PrL to the NAcc Core pathway may mediate drug craving and habit formation, while an opposing pathway from the IL to the NAcc Shell may act as a brake on drug and alcohol intake, by exerting inhibitory control over this Go pathway (Peters et al., 2008; LaLumiere et al., 2012; Meinhardt et al., 2013; Ma et al., 2014; Koob and Volkow, 2016; George and Hope, 2017). In this framework, we might interpret our results as a loss of control over drinking caused by an impaired ability of PACAP projections to the NAcc Shell to inhibit alcohol drinking. This relevant upstream source may be the IL, as PACAP is abundantly expressed there (Zhang et al., 2021), but further studies would be required to confirm this hypothesis.

The exact mechanism whereby functional deletion of PAC1R in the NAcc Shell leads to an increase in alcohol intake is unknown. The PACAP/PAC1R has been shown to affect glutamatergic and GABAergic signaling in other brain areas, through pre- or post-synaptic mechanisms (Macdonald et al., 2005; Costa et al., 2009; Varodayan et al., 2020). PACAP is also capable of exerting effects on neuronal excitability in the absence of glutamatergic or GABAergic input (May et al., 2021). This highlights the need for follow-up electrophysiological studies to further understand the specific actions of PAC1R activation in the NAcc Shell.

PACAP has two isoforms, PACAP-38, which represents the vast majority of PACAP in the brain, and PACAP-27 (Miyata et al., 1989; Piggins et al., 1996). Our data are in agreement with a recent report showing that administration of the peptide agonist PACAP-27 in the NAcc Shell decreases ethanol drinking in Long-Evans rats, while PACAP(6–27) antagonism of PAC1R acutely increases ethanol drinking (Gargiulo et al., 2021). Interestingly though, in that study, PACAP-38 and PACAP(6–38) had no effect when infused in the NAcc Shell, suggesting that the increase in alcohol drinking following knockdown of PAC1R we observed here may reflect a PACAP-27 dependent mechanism, although PACAP-38 is known to represent over 90% of the total PACAP present in the brain (Piggins et al., 1996). Further, this previously published study has suggested that the sub-region of the NAcc Shell along the rostro-caudal axis targeted is important for the effects of PACAP, as PACAP administration in the rostral shell decreased ethanol drinking, while in the caudal shell it increased drinking (Gargiulo et al., 2021). In our study, the AAV-PAC1R KD virus was targeted to the rostral portion of the NAcc, therefore our findings are in line with this previous report. We add to this previous study by showing a PAC1R-dependent mechanism, as the antagonists used above [PACAP(6–27), PACAP(6–38)] have been shown not to be selective for PAC1R, as they also bind VPAC2R (Dickinson et al., 1997). Indeed, RNA interference is a very useful technique for long term tissue-specific knockdown of precise targets, and it also has potential for translational use in the treatment of human disease (Shan, 2010; Takizawa et al., 2010). Importantly, the AAV-PAC1R-KD virus used in this study leads to a knockdown of all PAC1R isoforms (Blechman and Levkowitz, 2013), as different isoform expression has been proposed to fluctuate over the course of alcohol use (Gargiulo et al., 2020). Future studies focused on the expression of specific PAC1R variants in alcohol-preferring and wildtype animals prior to and during ethanol exposure are of great interest.

One limitation of this study is that the experiments were performed solely on male rats. Interestingly, it has been shown that there are no differences in lever-pressing between male and female sP rats (Lorrai et al., 2019) and no sex differences in the effect of PACAP administration in the NAcc Shell on home cage alcohol drinking in Long-Evans rats (Gargiulo et al., 2021). However, future studies will have to be performed in female animals, considering the sex differences seen in NAcc physiology and morphology, as well as those in alcohol drinking (Kovanen et al., 2010; Dragan et al., 2017; Liu et al., 2020; Radke et al., 2021; Townsley et al., 2021).

Overall, this study provides evidence that the loss of function of PAC1R in the NAcc Shell leads to excessive drinking and heightened motivation to drink, and therefore suggests that the PACAP/PAC1R system in this area may work as a “brake” on excessive alcohol drinking.

## Data Availability Statement

The original contributions presented in the study are included in the article/supplementary material, further inquiries can be directed to the corresponding author.

## Ethics Statement

The animal study was reviewed and approved by the Institutional Animal Care and Use Committee of the Boston University Medical Campus.

## Author Contributions

MM, VS, and PC designed the experiments. MM, TP, and ME performed the experiments. MM and VS analyzed the data. MM wrote a first draft of the manuscript. All authors edited the manuscript and approved the final version.

## Author Disclaimer

This article’s contents are solely the responsibility of the authors and do not necessarily represent the official views of the National Institutes of Health.

## Conflict of Interest

The authors declare that the research was conducted in the absence of any commercial or financial relationships that could be construed as a potential conflict of interest.

## Publisher’s Note

All claims expressed in this article are solely those of the authors and do not necessarily represent those of their affiliated organizations, or those of the publisher, the editors and the reviewers. Any product that may be evaluated in this article, or claim that may be made by its manufacturer, is not guaranteed or endorsed by the publisher.

## Footnotes

1
http://rgd.mcw.edu/rgdweb/report/strain/main.html?id=2302666

## References

[B1] Aschauer D. F. Kreuz S. Rumpel S. (2013). Analysis of transduction efficiency, tropism and axonal transport of AAV serotypes 1, 2, 5, 6, 8 and 9 in the mouse brain. *PLoS One* 8:e76310. 10.1371/journal.pone.0076310 24086725 PMC3785459

[B2] Barker J. M. Corbit L. H. Robinson D. L. Gremel C. M. Gonzales R. A. Chandler L. J. (2015). Corticostriatal circuitry and habitual ethanol seeking. *Alcohol* 49 817–824. 26059221 10.1016/j.alcohol.2015.03.003 PMC4644517

[B3] Bell R. L. Rodd Z. A. Lumeng L. Murphy J. M. McBride W. J. (2006). The alcohol-preferring P rat and animal models of excessive alcohol drinking. *Addict. Biol.* 11 270–288. 10.1111/j.1369-1600.2005.00029.x 16961759

[B4] Benvenuti F. Cannella N. Stopponi S. Soverchia L. Ubaldi M. Lunerti V. (2021). Effect of glucocorticoid receptor antagonism on alcohol self-administration in genetically-selected marchigian sardinian alcohol-preferring and non-preferring wistar rats. *Int. J. Mol. Sci.* 22:4184. 10.3390/ijms22084184 33920737 PMC8073469

[B5] Blasio A. Valenza M. Iyer M. R. Rice K. C. Steardo L. Hayashi T. (2015). Sigma-1 receptor mediates acquisition of alcohol drinking and seeking behavior in alcohol-preferring rats. *Behav. Brain Res.* 287 315–322. 10.1016/j.bbr.2015.03.065 25848705 PMC4424067

[B6] Blechman J. Levkowitz G. (2013). Alternative splicing of the Pituitary Adenylate Cyclase-Activating Polypeptide Receptor PAC1: mechanisms of fine tuning of brain activity. *Front. Endocrinol.* 4:55. 10.3389/fendo.2013.00055 23734144 PMC3659299

[B7] Cauvin A. Robberecht P. De Neef P. Gourlet P. Vandermeers A. Vandermeers-Piret M. C. (1991). Properties and distribution of receptors for pituitary adenylate cyclase activating peptide (PACAP) in rat brain and spinal cord. *Regul. Pept.* 35 161–173. 10.1016/0167-0115(91)90478-y 1661904

[B8] Ciccocioppo R. Hyytia P. (2006). The genetic of alcoholism: learning from 50 years of research. *Addict. Biol.* 11 193–194. 16961757 10.1111/j.1369-1600.2006.00028.x

[B9] Colombo G. Lobina C. Carai M. A. Gessa G. L. (2006). Phenotypic characterization of genetically selected Sardinian alcohol-preferring (sP) and -non-preferring (sNP) rats. *Addict. Biol.* 11 324–338. 10.1111/j.1369-1600.2006.00031.x 16961762

[B10] Costa L. Santangelo F. Li Volsi G. Ciranna L. (2009). Modulation of AMPA receptor-mediated ion current by pituitary adenylate cyclase-activating polypeptide (PACAP) in CA1 pyramidal neurons from rat hippocampus. *Hippocampus* 19 99–109. 10.1002/hipo.20488 18727050

[B11] Cox J. Witten I. B. (2019). Striatal circuits for reward learning and decision-making. *Nat. Rev. Neurosci.* 20 482–494. 10.1038/s41583-019-0189-2 31171839 PMC7231228

[B12] Crabbe J. C. (2014). Rodent models of genetic contributions to motivation to abuse alcohol. *Nebr. Symp. Motiv.* 61 5–29. 10.1007/978-1-4939-0653-6_2 25306777 PMC4988659

[B13] Dickinson T. Fleetwood-Walker S. M. Mitchell R. Lutz E. M. (1997). Evidence for roles of vasoactive intestinal polypeptide (VIP) and pituitary adenylate cyclase activating polypeptide (PACAP) receptors in modulating the responses of rat dorsal horn neurons to sensory inputs. *Neuropeptides* 31 175–185. 10.1016/s0143-4179(97)90087-1 9179871

[B14] Dore R. Iemolo A. Smith K. L. Wang X. Cottone P. Sabino V. (2013). CRF mediates the anxiogenic and anti-rewarding, but not the anorectic effects of PACAP. *Neuropsychopharmacology* 38 2160–2169. 10.1038/npp.2013.113 23657440 PMC3773665

[B15] Dragan W. L. Czerski P. M. Dragan M. (2017). PAC1 receptor (ADCYAP1R1) genotype and problematic alcohol use in a sample of young women. *Neuropsychiatr. Dis. Treat.* 13 1483–1489. 10.2147/NDT.S137331 28652748 PMC5473483

[B16] Ericson M. Blomqvist O. Engel J. A. Söderpalm B. (1998). Voluntary ethanol intake in the rat and the associated accumbal dopamine overflow are blocked by ventral tegmental mecamylamine. *Eur. J. Pharmacol.* 358 189–196. 10.1016/s0014-2999(98)00602-5 9822883

[B17] Ferragud A. Velazquez-Sanchez C. Minnig M. A. Sabino V. Cottone P. (2021). Pituitary adenylate cyclase-activating polypeptide (PACAP) modulates dependence-induced alcohol drinking and anxiety-like behavior in male rats. *Neuropsychopharmacology* 46 509–518. 10.1038/s41386-020-00904-4 33191400 PMC8027820

[B18] Gargiulo A. T. Curtis G. R. Barson J. R. (2020). Pleiotropic pituitary adenylate cyclase-activating polypeptide (PACAP): novel insights into the role of PACAP in eating and drug intake. *Brain Res.* 1729:146626. 10.1016/j.brainres.2019.146626 31883848 PMC6953419

[B19] Gargiulo A. T. Pirino B. E. Curtis G. R. Barson J. R. (2021). Effects of pituitary adenylate cyclase-activating polypeptide isoforms in nucleus accumbens subregions on ethanol drinking. *Addict. Biol.* 26:e12972. 10.1111/adb.12972 33020973 PMC8019681

[B20] George O. Hope B. T. (2017). Cortical and amygdalar neuronal ensembles in alcohol seeking, drinking and withdrawal. *Neuropharmacology* 122 107–114. 10.1016/j.neuropharm.2017.04.031 28435008 PMC5512851

[B21] Gessa G. L. (2016). The long pursued Holy Grail of the true “alcoholic” rat. *Brain Res.* 1645 55–57. 10.1016/j.brainres.2016.02.003 26867703

[B22] Ghatei M. A. Takahashi K. Suzuki Y. Gardiner J. Jones P. M. Bloom S. R. (1993). Distribution, molecular characterization of pituitary adenylate cyclase-activating polypeptide and its precursor encoding messenger RNA in human and rat tissues. *J. Endocrinol.* 136 159–166. 8094091 10.1677/joe.0.1360159

[B23] Gilpin N. W. Richardson H. N. Koob G. F. (2008). Effects of CRF1-receptor and opioid-receptor antagonists on dependence-induced increases in alcohol drinking by alcohol-preferring (P) rats. *Alcohol. Clin. Exp. Res.* 32 1535–1542. 10.1111/j.1530-0277.2008.00745.x 18631323 PMC2583093

[B24] Gray S. L. Yamaguchi N. Vencová P. Sherwood N. M. (2002). Temperature-sensitive phenotype in mice lacking pituitary adenylate cyclase-activating polypeptide. *Endocrinology* 143 3946–3954. 10.1210/en.2002-220401 12239106

[B25] Gupta A. Gargiulo A. T. Curtis G. R. Badve P. S. Pandey S. Barson J. R. (2018). Pituitary adenylate cyclase-activating Polypeptide-27 (PACAP-27) in the thalamic paraventricular nucleus is stimulated by ethanol drinking. *Alcohol. Clin. Exp. Res.* 42 1650–1660. 10.1111/acer.13826 29969146 PMC6120804

[B26] Hammack S. E. May V. (2015). Pituitary adenylate cyclase activating polypeptide in stress-related disorders: data convergence from animal and human studies. *Biol. Psychiatry* 78 167–177. 10.1016/j.biopsych.2014.12.003 25636177 PMC4461555

[B27] Hammack S. E. Cheung J. Rhodes K. M. Schutz K. C. Falls W. A. Braas K. M. (2009). Chronic stress increases pituitary adenylate cyclase-activating peptide (PACAP) and brain-derived neurotrophic factor (BDNF) mRNA expression in the bed nucleus of the stria terminalis (BNST): roles for PACAP in anxiety-like behavior. *Psychoneuroendocrinology* 34 833–843. 10.1016/j.psyneuen.2008.12.013 19181454 PMC2705919

[B28] Hammack S. E. Roman C. W. Lezak K. R. Kocho-Shellenberg M. Grimmig B. Falls W. A. (2010). Roles for pituitary adenylate cyclase-activating peptide (PACAP) expression and signaling in the bed nucleus of the stria terminalis (BNST) in mediating the behavioral consequences of chronic stress. *J. Mol. Neurosci.* 42 327–340. 10.1007/s12031-010-9364-7 20405238 PMC2955825

[B29] He D. Y. Vagts A. J. Yaka R. Ron D. (2002). Ethanol induces gene expression via nuclear compartmentalization of receptor for activated C kinase 1. *Mol. Pharmacol.* 62 272–280. 10.1124/mol.62.2.272 12130678

[B30] Heidbreder C. A. Groenewegen H. J. (2003). The medial prefrontal cortex in the rat: evidence for a dorso-ventral distinction based upon functional and anatomical characteristics. *Neurosci. Biobehav. Rev.* 27 555–579. 10.1016/j.neubiorev.2003.09.003 14599436

[B31] Heyser C. J. Schulteis G. Koob G. F. (1997). Increased ethanol self-administration after a period of imposed ethanol deprivation in rats trained in a limited access paradigm. *Alcohol. Clin. Exp. Res.* 21 784–791. 10.1111/j.1530-0277.1997.tb03840.x 9267526

[B32] Hurley M. M. Maunze B. Block M. E. Frenkel M. M. Reilly M. J. Kim E. (2016). Pituitary adenylate-cyclase activating polypeptide regulates hunger- and palatability-induced binge eating. *Front. Neurosci.* 10:383. 10.3389/fnins.2016.00383 27597817 PMC4993128

[B33] Hurley M. M. Robble M. R. Callan G. Choi S. Wheeler R. A. (2019). Pituitary adenylate cyclase-activating polypeptide (PACAP) acts in the nucleus accumbens to reduce hedonic drive. *Int. J. Obes.* 43 928–932. 10.1038/s41366-018-0154-6 30082747 PMC6363914

[B34] Iemolo A. Ferragud A. Cottone P. Sabino V. (2015). Pituitary adenylate cyclase-activating peptide in the central amygdala causes anorexia and body weight loss via the melanocortin and the TrkB systems. *Neuropsychopharmacology* 40 1846–1855. 10.1038/npp.2015.34 25649277 PMC4839508

[B35] Inglott M. A. Farnham M. M. Pilowsky P. M. (2011). Intrathecal PACAP-38 causes prolonged widespread sympathoexcitation via a spinally mediated mechanism and increases in basal metabolic rate in anesthetized rat. *Am. J. Physiol. Heart Circ. Physiol.* 300 H2300–H2307. 10.1152/ajpheart.01052.2010 21460201

[B36] Koob G. F. Volkow N. D. (2010). Neurocircuitry of addiction. *Neuropsychopharmacology* 35 217–238. 19710631 10.1038/npp.2009.110 PMC2805560

[B37] Koob G. F. Volkow N. D. (2016). Neurobiology of addiction: a neurocircuitry analysis. *Lancet Psychiatry* 3 760–773. 10.1016/S2215-0366(16)00104-8 27475769 PMC6135092

[B38] Kovanen L. Saarikoski S. T. Haukka J. Pirkola S. Aromaa A. Lonnqvist J. (2010). Circadian clock gene polymorphisms in alcohol use disorders and alcohol consumption. *Alcohol Alcohol.* 45 303–311. 10.1093/alcalc/agq035 20554694

[B39] Köves K. Arimura A. Görcs T. G. Somogyvári-Vigh A. (1991). Comparative distribution of immunoreactive pituitary adenylate cyclase activating polypeptide and vasoactive intestinal polypeptide in rat forebrain. *Neuroendocrinology* 54 159–169. 10.1159/000125864 1766552

[B40] Kwok C. Lei K. Pedrozo V. Anderson L. Ghotra S. Walsh M. (2021). Differential importance of nucleus accumbens Ox1Rs and AMPARs for female and male mouse binge alcohol drinking. *Sci. Rep.* 11:231. 10.1038/s41598-020-79935-2 33420199 PMC7794293

[B41] LaLumiere R. T. Smith K. C. Kalivas P. W. (2012). Neural circuit competition in cocaine-seeking: roles of the infralimbic cortex and nucleus accumbens shell. *Eur. J. Neurosci.* 35 614–622. 10.1111/j.1460-9568.2012.07991.x 22321070 PMC3281521

[B42] Liu Y. Montgomery S. E. Juarez B. Morel C. Zhang S. Kong Y. (2020). Different adaptations of dopamine release in nucleus accumbens shell and core of individual alcohol drinking groups of mice. *Neuropharmacology* 175:108176. 10.1016/j.neuropharm.2020.108176 32497591 PMC7492398

[B43] Lorrai I. Contini A. Gessa G. L. Mugnaini C. Corelli F. Colombo G. (2019). Operant, oral alcohol self-administration: sex differences in Sardinian alcohol-preferring rats. *Alcohol* 79 147–162. 10.1016/j.alcohol.2019.04.003 31029630

[B44] Lutfy K. Shankar G. (2019). Emerging evidence for the role of pituitary adenylate cyclase-activating peptide in neuropsychiatric disorders. *Prog. Mol. Biol. Transl. Sci.* 167 143–157. 10.1016/bs.pmbts.2019.06.009 31601402

[B45] Ma L. Chen W. Yu D. Han Y. (2020). Brain-wide mapping of afferent inputs to accumbens nucleus core subdomains and accumbens nucleus subnuclei. *Front. Syst. Neurosci.* 14:15. 10.3389/fnsys.2020.00015 32317941 PMC7150367

[B46] Ma Y. Y. Lee B. R. Wang X. Guo C. Liu L. Cui R. (2014). Bidirectional modulation of incubation of cocaine craving by silent synapse-based remodeling of prefrontal cortex to accumbens projections. *Neuron* 83 1453–1467. 10.1016/j.neuron.2014.08.023 25199705 PMC4295617

[B47] Macdonald D. S. Weerapura M. Beazely M. A. Martin L. Czerwinski W. Roder J. C. (2005). Modulation of NMDA receptors by pituitary adenylate cyclase activating peptide in CA1 neurons requires G alpha q, protein kinase C, and activation of Src. *J. Neurosci.* 25 11374–11384. 10.1523/JNEUROSCI.3871-05.2005 16339032 PMC6725893

[B48] Masuo Y. Suzuki N. Matsumoto H. Tokito F. Matsumoto Y. Tsuda M. (1993). Regional distribution of pituitary adenylate cyclase activating polypeptide (PACAP) in the rat central nervous system as determined by sandwich-enzyme immunoassay. *Brain Res.* 602 57–63. 10.1016/0006-8993(93)90241-e 8095427

[B49] May V. Johnson G. C. Hammack S. E. Braas K. M. Parsons R. L. (2021). PAC1 receptor internalization and endosomal MEK/ERK activation is essential for PACAP-mediated neuronal excitability. *J. Mol. Neurosci.* 71 1536–1542. 10.1007/s12031-021-01821-x 33675454 PMC8450765

[B50] Meinhardt M. W. Hansson A. C. Perreau-Lenz S. Bauder-Wenz C. Stählin O. Heilig M. (2013). Rescue of infralimbic mGluR2 deficit restores control over drug-seeking behavior in alcohol dependence. *J. Neurosci.* 33 2794–2806. 10.1523/JNEUROSCI.4062-12.2013 23407939 PMC3711176

[B51] Miyata A. Arimura A. Dahl R. R. Minamino N. Uehara A. Jiang L. (1989). Isolation of a novel 38 residue-hypothalamic polypeptide which stimulates adenylate cyclase in pituitary cells. *Biochem. Biophys. Res. Commun.* 164 567–574. 10.1016/0006-291x(89)91757-9 2803320

[B52] Moore M. S. DeZazzo J. Luk A. Y. Tully T. Singh C. M. Heberlein U. (1998). Ethanol intoxication in Drosophila: genetic and pharmacological evidence for regulation by the cAMP signaling pathway. *Cell* 93 997–1007. 10.1016/s0092-8674(00)81205-2 9635429

[B53] Musatov S. Chen W. Pfaff D. W. Kaplitt M. G. Ogawa S. (2006). RNAi-mediated silencing of estrogen receptor {alpha} in the ventromedial nucleus of hypothalamus abolishes female sexual behaviors. *Proc. Natl. Acad. Sci. U S A* 103 10456–10460. 10.1073/pnas.0603045103 16803960 PMC1502479

[B54] Mustafa T. Jiang S. Z. Eiden A. M. Weihe E. Thistlethwaite I. Eiden L. E. (2015). Impact of PACAP and PAC1 receptor deficiency on the neurochemical and behavioral effects of acute and chronic restraint stress in male C57BL/6 mice. *Stress* 18 408–418. 10.3109/10253890.2015.1025044 25853791 PMC4834918

[B55] Ongür D. Price J. L. (2000). The organization of networks within the orbital and medial prefrontal cortex of rats, monkeys and humans. *Cereb. Cortex* 10 206–219. 10.1093/cercor/10.3.206 10731217

[B56] Palkovits M. Somogyvari-Vigh A. Arimura A. (1995). Concentrations of pituitary adenylate cyclase activating polypeptide (PACAP) in human brain nuclei. *Brain Res.* 699 116–120. 10.1016/0006-8993(95)00869-r 8616598

[B57] Paxinos G. Watson C. (2007). *The Rat Brain in Stereotaxic Coordinates*, 6th edn. Amsterdam: Elsevier.

[B58] Peters J. LaLumiere R. T. Kalivas P. W. (2008). Infralimbic prefrontal cortex is responsible for inhibiting cocaine seeking in extinguished rats. *J. Neurosci.* 28 6046–6053. 10.1523/JNEUROSCI.1045-08.2008 18524910 PMC2585361

[B59] Piggins H. D. Stamp J. A. Burns J. Rusak B. Semba K. (1996). Distribution of pituitary adenylate cyclase activating polypeptide (PACAP) immunoreactivity in the hypothalamus and extended amygdala of the rat. *J. Comp. Neurol.* 376 278–294. 10.1002/(SICI)1096-9861(19961209)376:2<278::AID-CNE9>3.0.CO;2-0 8951643

[B60] Radke A. K. Sneddon E. A. Frasier R. M. Hopf F. W. (2021). Recent perspectives on sex differences in compulsion-like and binge alcohol drinking. *Int. J. Mol. Sci.* 22:3788. 10.3390/ijms22073788 33917517 PMC8038761

[B61] Reilly M. T. Noronha A. Goldman D. Koob G. F. (2017). Genetic studies of alcohol dependence in the context of the addiction cycle. *Neuropharmacology* 122 3–21. 10.1016/j.neuropharm.2017.01.017 28118990 PMC6233301

[B62] Reimsnider S. Manfredsson F. P. Muzyczka N. Mandel R. J. (2007). Time course of transgene expression after intrastriatal pseudotyped rAAV2/1, rAAV2/2, rAAV2/5, and rAAV2/8 transduction in the rat. *Mol. Ther.* 15 1504–1511. 10.1038/sj.mt.6300227 17565350

[B63] Resch J. M. Boisvert J. P. Hourigan A. E. Mueller C. R. Yi S. S. Choi S. (2011). Stimulation of the hypothalamic ventromedial nuclei by pituitary adenylate cyclase-activating polypeptide induces hypophagia and thermogenesis. *Am. J. Physiol. Regul. Integr. Comp. Physiol.* 301 R1625–R1634. 10.1152/ajpregu.00334.2011 21957159 PMC3233848

[B64] Resch J. M. Maunze B. Phillips K. A. Choi S. (2014). Inhibition of food intake by PACAP in the hypothalamic ventromedial nuclei is mediated by NMDA receptors. *Physiol Behav.* 133 230–235. 10.1016/j.physbeh.2014.05.029 24878316 PMC4126770

[B65] Ressler K. J. Mercer K. B. Bradley B. Jovanovic T. Mahan A. Kerley K. (2011). Post-traumatic stress disorder is associated with PACAP and the PAC1 receptor. *Nature* 470 492–497. 21350482 10.1038/nature09856 PMC3046811

[B66] Rezvani A. H. Overstreet D. H. Levin E. D. Rosenthal D. I. Kordik C. P. Reitz A. B. (2007). Effects of atypical anxiolytic N-phenyl-2-[1-[3-(2-pyridinylethynyl)benzoyl]-4-piperidine]acetamide (JNJ-5234801) on alcohol intake in alcohol-preferring P rats. *Alcohol. Clin. Exp. Res.* 31 57–63. 10.1111/j.1530-0277.2006.00264.x 17207102

[B67] Rodd Z. A. Bell R. L. Kuc K. A. Murphy J. M. Lumeng L. Li T. K. (2003). Effects of repeated alcohol deprivations on operant ethanol self-administration by alcohol-preferring (P) rats. *Neuropsychopharmacology* 28 1614–1621. 10.1038/sj.npp.1300214 12799615

[B68] Ross R. A. Hoeppner S. S. Hellberg S. N. O’Day E. B. Rosencrans P. L. Ressler K. J. (2020). Circulating PACAP peptide and PAC1R genotype as possible transdiagnostic biomarkers for anxiety disorders in women: a preliminary study. *Neuropsychopharmacology* 45 1125–1133. 10.1038/s41386-020-0604-4 31910434 PMC7235237

[B69] Ross R. A. Leon S. Madara J. C. Schafer D. Fergani C. Maguire C. A. (2018). PACAP neurons in the ventral premammillary nucleus regulate reproductive function in the female mouse. *Elife* 7:e35960. 10.7554/eLife.35960 29905528 PMC6013253

[B70] Sabino V. Cottone P. Blasio A. Iyer M. R. Steardo L. Rice K. C. (2011). Activation of sigma-receptors induces binge-like drinking in Sardinian alcohol-preferring rats. *Neuropsychopharmacology* 36 1207–1218. 10.1038/npp.2011.5 21346735 PMC3079320

[B71] Sabino V. Cottone P. Koob G. F. Steardo L. Lee M. J. Rice K. C. (2006). Dissociation between opioid and CRF1 antagonist sensitive drinking in Sardinian alcohol-preferring rats. *Psychopharmacology* 189 175–186. 10.1007/s00213-006-0546-5 17047935

[B72] Sabino V. Cottone P. Steardo L. Schmidhammer H. Zorrilla E. P. (2007). 14-Methoxymetopon, a highly potent mu opioid agonist, biphasically affects ethanol intake in Sardinian alcohol-preferring rats. *Psychopharmacology* 192 537–546. 10.1007/s00213-007-0746-7 17345066

[B73] Sabino V. Cottone P. Zhao Y. Iyer M. R. (2009). The sigma-receptor antagonist BD-1063 decreases ethanol intake and reinforcement in animal models of excessive drinking. *Neuropsychopharmacology* 34 1482–1493. 10.1038/npp.2008.192 18946467 PMC2669694

[B74] Sabino V. Kwak J. Rice K. C. Cottone P. (2013a). Pharmacological characterization of the 20% alcohol intermittent access model in Sardinian alcohol-preferring rats: a model of binge-like drinking. *Alcohol. Clin. Exp. Res.* 37 635–643. 10.1111/acer.12008 23126554 PMC3567206

[B75] Sabino V. Narayan A. R. Zeric T. Steardo L. Cottone P. (2013b). mTOR activation is required for the anti-alcohol effect of ketamine, but not memantine, in alcohol-preferring rats. *Behav. Brain Res.* 247 9–16. 10.1016/j.bbr.2013.02.030 23466691 PMC3646912

[B76] Scheimann J. R. Moloney R. D. Mahbod P. Morano R. L. Fitzgerald M. Hoskins O. (2019). Conditional deletion of glucocorticoid receptors in rat brain results in sex-specific deficits in fear and coping behaviors. *Elife* 8:e44672. 10.7554/eLife.44672 31329100 PMC6645713

[B77] Schroeder J. P. Overstreet D. H. Hodge C. W. (2005). The mGluR5 antagonist MPEP decreases operant ethanol self-administration during maintenance and after repeated alcohol deprivations in alcohol-preferring (P) rats. *Psychopharmacology* 179 262–270. 10.1007/s00213-005-2175-9 15717208 PMC11583314

[B78] Seiglie M. P. Huang L. Cottone P. Sabino V. (2019). Role of the PACAP system of the extended amygdala in the acoustic startle response in rats. *Neuropharmacology* 160:107761. 10.1016/j.neuropharm.2019.107761 31493466 PMC6842120

[B79] Shan G. (2010). RNA interference as a gene knockdown technique. *Int. J. Biochem. Cell Biol.* 42 1243–1251. 10.1016/j.biocel.2009.04.023 19442757

[B80] Sherwood N. M. Krueckl S. L. McRory J. E. (2000). The origin and function of the pituitary adenylate cyclase-activating polypeptide (PACAP)/glucagon superfamily. *Endocr. Rev.* 21 619–670. 10.1210/edrv.21.6.0414 11133067

[B81] Shield K. Manthey J. Rylett M. Probst C. Wettlaufer A. Parry C. D. H. (2020). National, regional, and global burdens of disease from 2000 to 2016 attributable to alcohol use: a comparative risk assessment study. *Lancet Public Health* 5 e51–e61. 10.1016/S2468-2667(19)30231-2 31910980

[B82] Steensland P. Simms J. A. Holgate J. Richards J. K. Bartlett S. E. (2007). Varenicline, an alpha4beta2 nicotinic acetylcholine receptor partial agonist, selectively decreases ethanol consumption and seeking. *Proc. Natl. Acad. Sci. U S A* 104 12518–12523. 17626178 10.1073/pnas.0705368104 PMC1914040

[B83] Stojakovic A. Ahmad S. M. Malhotra S. Afzal Z. Ahmed M. Lutfy K. (2020). The role of pituitary adenylyl cyclase-activating polypeptide in the motivational effects of addictive drugs. *Neuropharmacology* 171:108109. 10.1016/j.neuropharm.2020.108109 32325064

[B84] Substance Abuse and Mental Health Services Administration (SAMHSA) (2019). *2019 National Survey on Drug Use and Health.* Rockvile, MD: SAMHSA.

[B85] Suchankova P. Steensland P. Fredriksson I. Engel J. A. Jerlhag E. (2013). Ghrelin receptor (GHS-R1A) antagonism suppresses both alcohol consumption and the alcohol deprivation effect in rats following long-term voluntary alcohol consumption. *PLoS One* 8:e71284. 10.1371/journal.pone.0071284 23977009 PMC3748070

[B86] Szabó G. Mácsai M. Schek E. Telegdy G. (1998). The effect of vasoactive intestinal polypeptide and pituitary adenylate cyclase activating polypeptide on tolerance to morphine and alcohol in mice. *Ann. N. Y. Acad. Sci.* 865 566–569. 10.1111/j.1749-6632.1998.tb11238.x 9928071

[B87] Takizawa T. Gemma A. Ui-Tei K. Aizawa Y. Sadovsky Y. Robinson J. M. (2010). Basic and clinical studies on functional RNA molecules for advanced medical technologies. *J. Nippon Med. Sch.* 77 71–79. 10.1272/jnms.77.71 20453418

[B88] Tanaka K. Hashimoto H. Shintani N. Yamamoto A. Baba A. (2004). Reduced hypothermic and hypnotic responses to ethanol in PACAP-deficient mice. *Regul. Pept.* 123 95–98. 10.1016/j.regpep.2004.05.017 15518898

[B89] Torruella-Suárez M. L. Vandenberg J. R. Cogan E. S. Tipton G. J. Teklezghi A. Dange K. (2020). Manipulations of central amygdala neurotensin neurons alter the consumption of ethanol and sweet fluids in mice. *J. Neurosci.* 40 632–647. 10.1523/JNEUROSCI.1466-19.2019 31744862 PMC6961987

[B90] Townsley K. G. Borrego M. B. Ozburn A. R. (2021). Effects of chemogenetic manipulation of the nucleus accumbens core in male C57BL/6J mice. *Alcohol* 91 21–27. 10.1016/j.alcohol.2020.10.005 33160072 PMC8675149

[B91] Valenza M. DiLeo A. Steardo L. Cottone P. Sabino V. (2016). Ethanol-related behaviors in mice lacking the sigma-1 receptor. *Behav. Brain Res.* 297 196–203. 10.1016/j.bbr.2015.10.013 26462569 PMC4679530

[B92] Varodayan F. P. Minnig M. A. Steinman M. Q. Oleata C. S. Riley M. W. Sabino V. (2020). PACAP regulation of central amygdala GABAergic synapses is altered by restraint stress. *Neuropharmacology* 168:107752. 10.1016/j.neuropharm.2019.107752 31476352 PMC7048635

[B93] Vengeliene V. Siegmund S. Singer M. V. Sinclair J. D. Li T. K. Spanagel R. (2003). A comparative study on alcohol-preferring rat lines: effects of deprivation and stress phases on voluntary alcohol intake. *Alcohol. Clin. Exp. Res.* 27 1048–1054. 10.1097/01.ALC.0000075829.81211.0C 12878910

[B94] Walker B. M. Rasmussen D. D. Raskind M. A. Koob G. F. (2008). alpha1-noradrenergic receptor antagonism blocks dependence-induced increases in responding for ethanol. *Alcohol* 42 91–97. 10.1016/j.alcohol.2007.12.002 18358987 PMC2587143

[B95] You C. Savarese A. Vandegrift B. J. He D. Pandey S. C. Lasek A. W. (2019). Ethanol acts on KCNK13 potassium channels in the ventral tegmental area to increase firing rate and modulate binge-like drinking. *Neuropharmacology* 144 29–36. 10.1016/j.neuropharm.2018.10.008 30332606 PMC6286249

[B96] Zhang L. Hernandez V. S. Gerfen C. R. Jiang S. Z. Zavala L. Barrio R. A. (2021). Behavioral role of PACAP signaling reflects its selective distribution in glutamatergic and GABAergic neuronal subpopulations. *Elife* 10:e61718. 10.7554/eLife.61718 33463524 PMC7875564

[B97] Zinsmaier A. K. Dong Y. Huang Y. H. (2021). Cocaine-induced projection-specific and cell type-specific adaptations in the nucleus accumbens. *Mol. Psychiatry* Online ahead of print. 10.1038/s41380-021-01112-2 33963288 PMC8691189

